# Deprivation and well-being in squalid living: a propensity score matched cross-sectional study of the English Housing Survey

**DOI:** 10.1186/s12889-025-23440-6

**Published:** 2025-07-02

**Authors:** Mike Norton, Stephen Kellett, Vyv Huddy

**Affiliations:** 1 University of Sheffield, Sheffield, UK; 2 Rotherham Doncaster and South Humber NHS Trust, Doncaster, UK

**Keywords:** Squalor, Diogenes syndrome, Severe domestic squalor, Self-neglect, Well-being, Deprivation

## Abstract

**Background:**

There is a lack of understanding of people that live in severe domestic squalor (i.e., when their dwelling is grossly unclean/disorganised/unhygienic) and how they might differ from community controls. This study therefore sought to compare people living in squalor in terms of potential differences in deprivation and well-being.

**Methods:**

Data was extracted from the English Housing Survey. A sample of *N* = 298 people independently assessed as living in squalor from *N* = 43,222 household surveys were propensity score matched on seven demographic variables with *N* = 596 community controls. The two study groups were then compared on measures of deprivation and well-being and these variables were entered into regressions to predict living in squalor.

**Results:**

People living in squalor reside in significantly more deprived areas but are not significantly less satisfied/happy or significantly more anxious/worthless. An increase of 1 level on the deprivation scale decreased risk of living in squalor by 9%.

**Conclusions:**

Local deprivation appears to play a significant role in living in squalor. This may create ‘bi-directional causality’ in that local deprivation increases risk of squalor, and living in squalor adds to local deprivation. There needs to be more controlled research regarding squalor, so that targets for intervention (that are malleable) can then be identified, implemented and evaluated.

## Introduction

Snowdon et al. [1] stated that a person is living in Severe Domestic Squalor (SDS) when their home is “…so unclean, messy and unhygienic that people of a similar culture and background would consider extensive cleaning and clearing to be essential”. This situation is also commonly referred to in the literature as Diogenes Syndrome (DS) [2, 3]. A recent prevalence meta-analysis [4] estimated the point prevalence of squalor to be 0.85%. More significant local community deprivation, living in a rented property, lower income levels and high numbers of people in the dwelling were significant predictors of squalor in the subgroup analysis. Several other studies have also identified characteristics of individuals who live in squalor [5–12]. These studies suggest that people who live in squalor often live alone, commonly show comorbid hoarding behaviours and are likely to have a comorbid mental disorder or cognitive impairment such as dementia, psychotic disorders, alcohol misuse and executive dysfunction.

The social and physical consequences of self and environmental neglect, as occur in squalor and related conditions, are also extensive. Social isolation is common, as individuals are often judged negatively for the state of their property and their own personal hygiene, which leads to them not allowing people into their home and reducing contact with others socially [13, 14]. From a safety perspective, a build-up of materials in the home increases the risk of fires [15–17] and of trips and falls [18, 19]. Furthermore, individuals who neglect their environment are shown to have poorer health than those who do not [20] and be at risk of physical illness comorbidities such as asthma, dermatitis, infections [21], anaemia, dehydration and mineral/vitamin deficiency [22].

There are a wide range of methodological difficulties with studies describing the characteristics of individuals living in squalor. Studies are frequently limited to people aged 65 years and older [10–12, 23] and often feature convenience sampling methods. Furthermore, there is a lack of detailed statistical methods and analyses [8, 23–25]. A key methodological weakness is the limited use of control and comparison groups, so there is little systematic evaluation of how people living in squalor differ from community controls. There are few findings of significant differences between older adults living in squalor with non-squalor controls [2, 5, 10]. The use of older samples limits generalisation of findings, particularly in terms of variables such as marital status and living alone.

The final area of methodological concern in the squalor literature is the over focus on the individual and the relative under focus on the context. Research has focused primarily on the characteristics of the person who is living in squalor, especially their cognitive profile. Local and household factors such as unemployment, socioeconomic status and ethnic composition have been neglected. These social determinants that contribute to the risk of psychiatric conditions have consistently been shown to be important [26, 27]. These include poverty, unemployment, and local factors such as social disorder and the neglected local built environment [27]. Local deprivation, in particular, has been identified as a key factor in creating and maintaining poor mental health [28, 29]. Deprivation encompasses a range of factors including income, employment, education, health and housing [30]. Research into self-neglect (SN), which includes individuals living in squalor, has shown a significant relationship with deprivation, such that SN is more common in deprived areas [31, 32]. If local deprivation is related to SN, this suggests that it could also be related to squalor, a more specific form of SN. Therefore, this may represent a new area of focus in squalor research and suggests that community level interventions could be considered.

Mental health is a significant issue in squalid living, with conditions such as dementia, alcohol abuse and psychotic disorders commonly identified [7, 11, 12, 24, 33]. Scores on measures of psychological well-being have been shown to be associated with dementia [34, 35], alcohol and substance misuse [36, 37] and psychosis [38–40], such that well-being is lower in those with more severe conditions. Therefore, scores on these well-being questions appear to be related to some of the most common mental health conditions identified in squalor. However, it should be noted that, unlike many populations, individuals living in squalor commonly show limited insight and awareness of professional evaluations of their living conditions [6, 8, 41]. In the case of squalor, if individuals do not consider their environment to be a problem, their subjective well-being may not be affected, compared to those who are not living in squalor.

Due to the isolated nature of squalor [13], involving individuals who live in these conditions in research is particularly difficult. Furthermore, many individuals do not engage with services [42], also making them unlikely to agree to participate in research. In addition, due to the low prevalence of squalor in the general population, a large survey is required to provide an appropriately sized sample for the study of individuals living in squalor. Therefore, it is not uncommon for squalor studies to use medical records, or other secondary datasets, to provide a sample which can be used to make significant inferences regarding the individuals in question [5, 8, 43]. However, previous squalor research has used datasets limited to older adults and has not considered squalor in the general population, which is the approach of the present study.

This study intends to identify a sample of individuals living in squalor and use propensity score matching (PSM) to create a non-squalor sample, matching on several demographic and household variables. PSM has not been used in squalor research before. The study will investigate two variables which have been identified as factors in related conditions but have not previously been considered in squalor research. Firstly, measuring the well-being of squalor and non-squalor individuals in the general population gives an opportunity to investigate what elements of well-being, if any, have a relationship with an individual’s living conditions. Secondly, by investigating deprivation, a new direction in squalor research is introduced to the field that focuses on the role of socioeconomic, rather than individual, factors and whether they are connected to an individual’s personal environment. Based on the rationale for the study, the following hypotheses are proposed: [1] the rate of living in squalor will be significantly higher in contexts of high local deprivation and [2] individuals with lower subjective well-being will be more likely to be living in squalor.

## Methods

### Ethics and data

The study was ethically reviewed and approved by the University of Sheffield as a secondary data analysis (ref: 049202) and complied with the requirements of the UK Data Service [44] regarding using their datasets for research purposes. The current study used data provided by the English Housing Survey (EHS; 45). The EHS is a continuous national UK survey, first conducted in 1967, that collects information about people’s housing circumstances, demographics and psychological well-being. The UK Statistics Authority states that the statistics in the EHS are “produced according to sound methods and managed impartially and objectively in the public interest.” [46]. Each year a sample of UK houses are drawn at random and invited to participate by letter. Those that agree to take part in a face-to-face interview survey are also invited to take part in the physical survey element of the study, where a qualified surveyor comes to the property and completes a visual inspection of the interior, exterior and local area. Consent is confirmed by the participant for the face-to-face interview and again for the physical survey. The EHS makes contact with a significant number of randomly-selected households each year with around 6,000 allowing their property to be subject to the physical inspection [45]. In this study, data from both the 2016 (Includes data from April 2015 to March 2017) and 2018 (Includes data from April 17 to March 19) datasets were investigated to identify individuals living in squalor [47, 48]. At the point of analysis, datasets closer to the present time were not available, as COVID stopped all in-person surveys.

### Measures

The English Housing Survey collects data on a wide range of variables, including characteristics of the individuals, their home and the local neighbourhood. This study included several variables from the survey, two of which operated as the main independent variables and one as a dependent variable. These were local deprivation, mental well-being of the individual and the presence of squalor, respectively.

### Local deprivation

The area surrounding the dwelling is scored for deprivation. Values range from 1 to 10, with a value of 1 showing that the property resides in one of the 10% most deprived locations and a value of 10 for properties in one of the 10% least deprived areas. All deprivation rankings used in the current study were based on the IMD ratings from 2015 [49].

### Subjective well-being

Four questions measured subjective well-being. Although a house may have multiple residents, well-being measures are only assessed for the individual who completes the face-to-face questionnaire. The four items are “Overall, how satisfied are you with your life, nowadays?”, “Overall, to what extent do you feel that the things you do in your life are worthwhile?”, “Overall, how happy did you feel yesterday?” and “On a scale where 0 is ‘not at all anxious’ and 10 is ‘completely anxious’, overall, how anxious did you feel yesterday?”. Each item is scored from 0 to 10. These four items make up the Office for National Statistics Subjective Well-Being Questions (ONS-4;) [50]. The ONS-4 are commonly used in community surveys and questionnaire studies [51] and have also been used in published research [52–54]. The ONS-4 is not a fully validated measure [53], but has been shown to have good internal reliability (α = 0.90) [55]. In the present study, across the whole dataset, the ONS-4 had an acceptable internal reliability (α = 0.75). Nonetheless, it is recommended that the questions are not aggregated as the items represent distinct conceptual domains [56]. Therefore, scores will be given for each item separately.

### Squalor

This was based on a measure from the physical survey of the property. The surveyor rated the risk due to ‘domestic hygiene, pests and refuse’ inside the property. Potential ratings were ‘significantly lower risk than average’, ‘average’, ‘significantly higher risk than average’ and ‘extreme’. In the physical survey data, over 99% of households were ranked as ‘average’. For the purposes of this study, any individual deemed to be at ‘significantly higher risk’ or ‘extreme’ risk were considered to be living in squalor.

### Supplementary variables

In addition to the main independent and dependent variables, data from additional factors was also collected. These were included as potential controlling variables or future areas of investigation: sex - male or female; age - values up to 85 (any individual 85 or older is given a value of 85); only individuals 18 or older will be included; gross household income– total annual income from both the individual and their partner, including state and housing benefits (ranges from £0 to £100000 and values greater than £100000 are given a value of £100000); tenure– the ownership status of the house (owner occupier, private rented, local authority or housing association); household type– this variable gives information as to who is present in the household (couple with or without children, lone parent, other multi-person household, one person (under 60 years old) or one person (60 years and older); ethnicity (White, Black/African/Caribbean/Black British, Indian, Pakistani and Bangladeshi, Other Asian, Chinese, Mixed/Multiple ethnic groups, other ethnic group). To remain consistent with the ethical requirements of the UK Data Service, this has been reduced to two categories in some sections of the analysis– ‘White’ and ‘Black, Asian and Minority Ethnic’ (BAME). General health– this question asks, “How is your health in general?” with 5 possible options (very bad, bad, fair, good, very good); long-standing disability or illness (any physical, mental health conditions or illness lasting 12 months or more? Options are yes, no, don’t know, or refuse to answer). Follow-up questions for those with a disability/illness include: Day-to-day activities - whether the condition affects ability to carry out day-to-day activities (Yes, a lot; Yes, a little; Not at all); nature of condition– does the condition affect you in the following areas; vision, hearing, mobility, dexterity, learning difficulties, memory, mental health, stamina, socially, other.

### Analysis

**Fig. 1:**
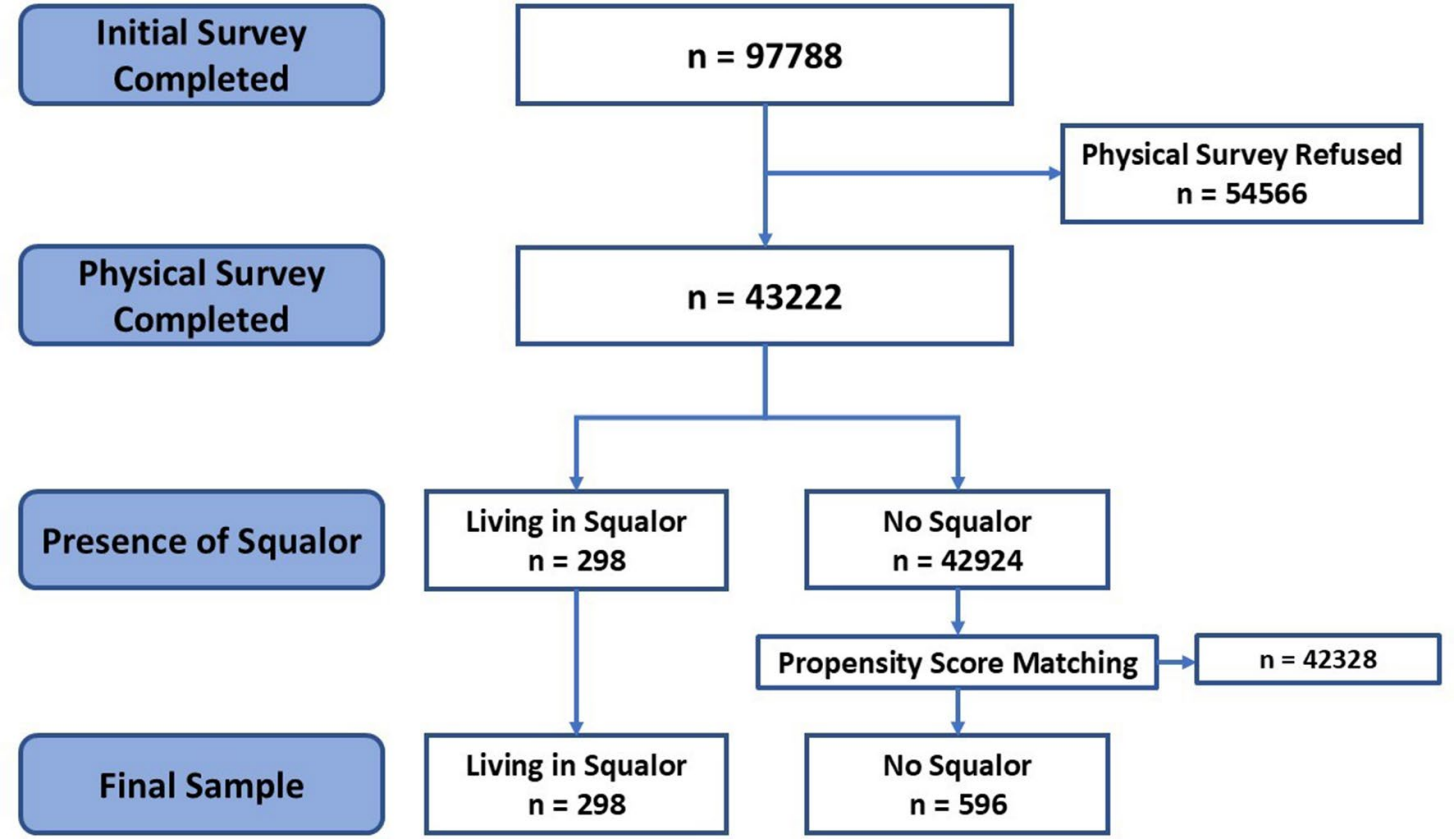
Flow chart showing the sample size at each stage of the data analysis process

All analysis was conducted using R, version 4.1.2. Fig. 1 details the process by which the data was reduced to form a squalor group and a control group. Once under-18s had been removed, the study included *n* = 97,788 adults. These were initially split into individuals who chose not to complete the physical survey and those that agreed for it to take place. The 43,222 adults who came from households which had agreed to the physical survey were then split into those who lived in squalor (*n* = 298) and those who were not classified as living in squalor (*n* = 42924). These two groups were compared using t-tests, chi-squared tests and effect sizes (Cohen’s d and Cramer’s V) to identify any significant differences between the two groups. To reduce the risk of type-1 errors, a Bonferroni correction [57] was applied directly to the p-values calculated by the t-tests. This allowed for an adjustment, while maintaining a significance value of *p* =.05.

PSM was used to identify a control sample which could be analysed alongside the squalor group. PSM creates a balanced dataset for comparison, resembling randomisation in clinical trials, so allowing for effective comparisons of variables of interest between the two groups [58]. In this study, this allowed a control group to be produced which matched the squalor group on key covariates. Given that the focus of the study was on the relationship between squalor and the two variables deprivation and well-being, PSM used all additional variables which had a complete set of data. This meant that the two study groups were matched by database, age, sex, general health, household income, whether they lived alone, whether they owned their house and number of people in the household. Using the nearest neighbour PSM proposed by Zhao et al. [59], the approach that produced the most balanced set of control group data was using a ratio of 2 non-squalor individuals for every squalor individual. It was not possible to match by ethnicity or specific illness data as the matching process required a complete dataset. Therefore, to complete this would have meant removing members of the squalor group, which was already significantly lower than the non-squalor group. Instead, these variables were included as controls in the regression analysis. The final squalor and non-squalor groups were compared using t-tests and chi-squared tests and effect sizes calculated. Furthermore, logistic regression was employed to assess the effect of the independent variables of deprivation and well-being and to control for ethnicity and illness. This analysis process allowed for the main independent variables of deprivation and well-being to be compared and investigated with a statistically robust comparison group, identifying significant differences and helping to understand whether these factors predict the likelihood of an individual living in a squalid home.

## Results

Table 1 shows the summarised data for the *n* = 43,222 individuals who had their homes surveyed.

**Table 1:** Summarised data of individuals who had their homes surveyed

Variable	Mean (SD)
Deprivation	5.04 (2.88)
Well-being– Satisfaction Worthwhile Happy Anxious	7.56 (1.97) 7.82 (1.89) 7.48 (2.24) 2.85 (2.96)
Age	48.26 (18.30)
Household size	2.77 (1.41)
Income	£35,084 (23160)
General health	3.97 (0.99)
Variable	% of sample
Sex (% Male)	47.2
Age (%) 18–19 20–29 30–39 40–49 50–59 60–69 70–79 80-	2.9 16.3 18.1 16.4 16.0 14.8 10.7 4.8
Ethnicity (%)– White Black+ Indian Pakistani+ Other Asian Chinese Mixed/Multiple Other White BAME	87.2 3.8 2.1 2.4 1.1 0.4 1.5 1.5 87.2 12.8
Tenure (%) - Owner Occupied Private Rented Local Authority (LA) Housing Association (HA) Owned Rented/LA/HA	45.6 20.6 14.2 19.5 45.6 54.4
Household type (%) - Couple, no children Couple + children Lone parent Other multi-person One person < 60 One person 60+ Living alone Living with others	38.5 26.1 6.5 12.2 6.8 9.8 16.6 83.4
Illness (% with illness)	35.2
Limitations due to illness (%) Not at all A little A lot	27.7 36.5 35.7
Type of limitations (% of ill with this limitation) - Vision Hearing Mobility Dexterity Learning Difficulties Memory Mental Health Stamina Social Other None	13.3 13.8 43.4 23.6 10.9 14.4 20.8 36.0 5.6 5.9 20.5

The sample of *n* = 43,222 individuals who allowed their home to be surveyed produced *n* = 298 who were found to be living in squalor and *n* = 42,924 who were not. The full details of these two groups can be seen in Table 2. These groups were not matched. However, the data suggests that there were several significant differences between those who were living in squalor and those who were not. This is the case in the levels of deprivation and three of the four measures of well-being, the main variables of interest. Furthermore, household income, household personnel and type of tenure also appeared to differ, such that squalid households are likely to have a lower total income, are more likely to be rented and are more likely to be a lone parent or an individual living alone.

**Table 2:** Comparison of individuals living in squalor and those not living in squalor

Variable	Living in squalor *N* = 298	No squalor *N* = 42,924	Significance	Effect size (Cohen’s/ Cramer’s)
Deprivation	3.70 (2.39)	5.05 (2.88)	*p* <.0001	*d* = 0.47
Well-being– Satisfaction Worthwhile Happy Anxious	6.99 (2.43) 7.43 (2.11) 6.96 (2.44) 3.52 (3.25)	7.56 (1.96) 7.82 (1.89) 7.48 (2.24) 2.84 (2.96)	*p* <.05 n.s. *p* <.05 *p* <.05	*d* = 0.29 *d* = 0.21 *d* = 0.23 *d* = 0.23
Age	46.03 (17.89)	48.27 (18.30)	n.s.	*d* = 0.12
Household size	2.88 (1.57)	2.77 (1.41)	n.s.	*d* = 0.08
Income	£25,488 (16966)	£35,151 (23184)	*p* <.0001	*d* = 0.42
General health	3.71 (1.12)	3.97 (0.99)	*p* <.01	*d* = 0.26
Age (%) 18–19 20–29 30–39 40–49 50–59 60–69 70–79 80-	4.7 18.8 16.4 17.8 17.8 12.4 9.7 2.3	2.9 16.3 18.1 16.4 16.0 14.8 10.7 4.8	n.s.	*v* = 0.02
Ethnicity (%)– White BAME	85.2 14.8	87.2 12.8	n.s.	*v* = 0.00
Sex (% Male)	53.0	47.2	n.s.	*v* = 0.01
Tenure (%) - Owner Occupied Private Rented Local Authority Housing Association Owned Rented/council/housing association	27.2 22.8 19.8 30.2 27.2 72.8	45.8 20.6 14.2 19.4 45.8 54.2	*p* <.0001 *p* <.0001	*v* = 0.03 *v* = 0.03
Household type (%) - Couple, no children Couple + children Lone parent Other multi-person One person < 60 One person 60+ Living alone Living with others	24.2 18.5 17.4 18.8 10.1 11.1 21.1 78.9	38.6 26.2 6.4 12.1 6.8 9.8 16.6 83.4	*p* <.0001 *p* <.05	*v* = 0.05 *v* = 0.01
Illness (% with illness)	40.9	35.2	*p* <.05	*v* = 0.01
Limitations due to illness (%) - Not at all A little A lot	20.7 35.5 43.8	27.8 36.5 35.7	n.s.	*v* = 0.02
Type of limitations (% of ill with this limitation) - Vision Hearing Mobility Dexterity Learning Difficulties Memory Mental Health Stamina Social Other None	14.9 15.7 47.9 26.4 9.9 16.5 33.1 32.2 8.3 7.4 18.2	13.3 13.8 43.4 23.5 10.9 14.4 20.7 36.0 5.6 5.9 20.5	n.s. n.s. n.s. n.s. n.s. n.s. *p* <.01 n.s. n.s. n.s. n.s.	*v* = 0.00 *v* = 0.00 *v* = 0.01 *v* = 0.01 *v* = 0.00 *v* = 0.00 *v* = 0.03 *v* = 0.01 *v* = 0.01 *v* = 0.00 *v* = 0.00

Note. Standard Deviation (SD) included in brackets

Following PSM, the final research sample included *n* = 298 individuals living in squalor and *n* = 596 who were not. The two groups were matched on databases (2016 or 2018), on age, sex, general health, household size, household income, whether they lived alone and whether they owned their own house. Table 3 shows the variables by which the two groups were matched and their average values following the PSM process. The standard mean difference (SMD) and variance ratio (VR) of the matched variables were between − 0.1 and 0.1, and between 0.5 and 2.0, respectively, showing them to be well-balanced [59].

**Table 3:** Comparison of the squalor and non-squalor group on the matched variables

Variable	Living in squalor *N* = 298	No squalor *N* = 596	Standard Mean Difference (SMD)	Variance Ratio (VR)
Database (%) 2016 2018	56.0 44.0	57.9 42.1	0.019	
Age	46.03	47.45	-0.080	0.948
Sex (% Male)	53.02	55.03	0.020	
General health	3.72	3.71	0.009	1.040
Income	25,488	25,428	0.004	1.190
Household size	2.88	2.86	0.016	0.916
Tenure (%) - Owned Rented/council/housing association	27.2 72.8	27.2 72.8	0.000	
Household type (%) - Living alone Living with others	21.1 78.9	21.3 78.7	0.002	

Note. -0.1 < SMD < 0.1 and 0.5 < VR < 2.0 suggests balanced data (Zhao et al., 2021)

The independent variables of interest were then compared between the matched squalor and non-squalor groups (see Table 4).

**Table 4:** Comparison of mean values for deprivation and well-being measures

Variable	Living in squalor *N* = 298	No squalor *N* = 596	Significance	Effect size (Cohen’s)
Deprivation	3.70 (2.39)	4.35 (2.75)	*p* <.001	*d* = 0.25
Well-being– Satisfaction Worthwhile Happy Anxious	6.99 (2.43) 7.43 (2.11) 6.96 (2.44) 3.52 (3.25)	7.14 (2.22) 7.42 (2.15) 7.12 (2.42) 3.30 (3.25)	n.s. n.s. n.s. n.s.	*d* = 0.07 *d* = 0.02 *d* = 0.07 *d* = 0.07

Note. Standard Deviation (SD) included in brackets

**Fig. 2:**
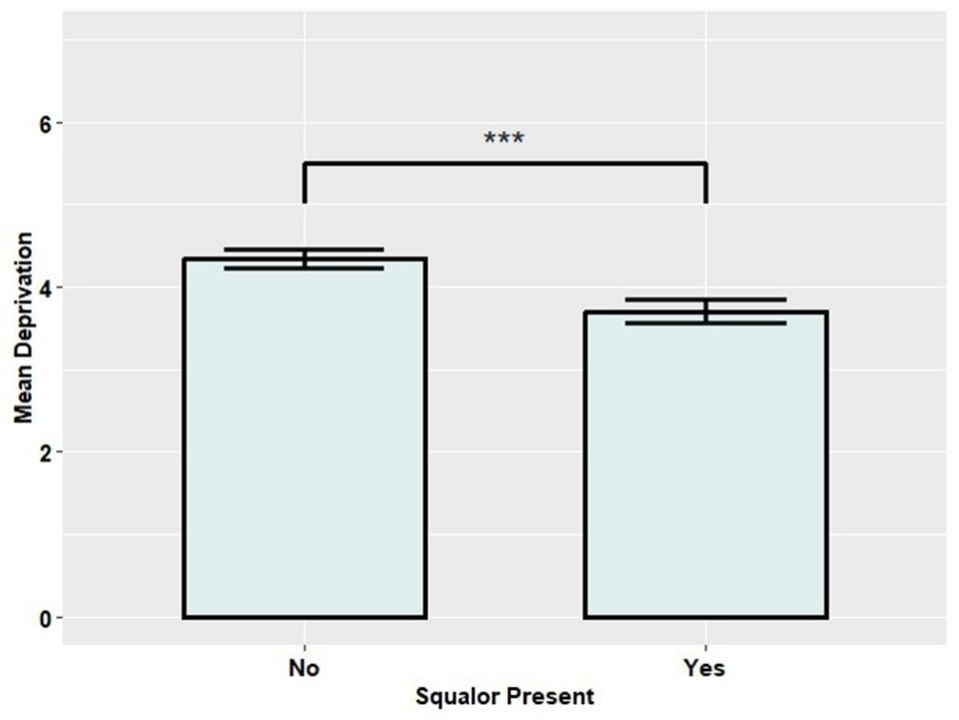
Bar chart showing the difference in mean deprivation score. *Note*. Error bars represent standard errors (SE)

In comparison to those not living in squalor (M = 4.35, SD = 2.75), individuals living in squalor (*M* = 3.70, *SD* = 2.39) were shown to reside in significantly more deprived areas (t(671.93) = -3.62, *p* <.001) - see Fig. 2. Conversely, none of the well-being measures showed a significant difference between the squalor (Satisfaction: M = 6.99, SD = 2.43; Worthwhile: M = 7.43, SD = 2.11; Happiness: M = 6.96, SD = 2.44; Anxiety: M = 3.52, SD = 3.25) and non-squalor groups (Satisfaction: M = 7.14, SD = 2.22; Worthwhile: M = 7.42, SD = 2.15; Happiness: M = 7.12, SD = 2.42; Anxiety: M = 3.30, SD = 3.25).

The results of the logistic regression (see Table 5) support the findings from the comparisons. Model 1 shows that deprivation is a significant predictor of whether an individual lives in squalor, such that an individual living in a more deprived area is more likely to be living in squalid conditions. In fact, the coefficient of the deprivation variable suggests that an increase of 1 level on the deprivation scale will decrease the risk of living in squalor by around 9%. Deprivation continued to be significant regardless of the inclusion of well-being or control variables. However, the significance level did adjust to *p* <.05 when other factors were added. Measures of well-being were shown to be poor predictors of an individual’s risk of living in squalor (Model 2) and this continued to be the case when included with deprivation and with the control variables of ethnicity and illness.

**Table 5:** Regression outcomes for the main variables and control variables

Variable	Model 1	Model 2	Model 3	Model 4
Intercept	-0.31 (0.13)*	-0.64 (0.47)	-0.42 (0.48)	-0.40 (0.63)
Deprivation	-0.10 (0.03)***		-0.09 (0.04)*	-0.10 (0.04)*
Well-being– Satisfaction		-0.03 (0.06)	-0.03 (0.06)	-0.04 (0.07)
Well-being - Worthwhile		0.03 (0.06)	0.04 (0.07)	0.03 (0.07)
Well-being– Happy		-0.02 (0.05)	-0.01 (0.05)	-0.00 (0.05)
Well-being– Anxious		0.01 (0.03)	0.02 (0.03)	0.03 (0.03)
Ethnicity				0.13 (0.30)
Illness				-0.29 (0.21)

Note. Standard Error (SE) included in brackets

* - *p* <.05, ** - *p* <.01, *** - *p* <.01

## Discussion

The aim of this study was to investigate whether there was a relationship between living in squalor and either the individual’s current mental well-being or deprivation in their local area. The study tapped a previously unused research resource, the English Housing Survey, generating a large and suitably powered sample. The study also used PSM to increase the precision of comparisons, as this method has not been used previously in the squalor field. Based on previous data into squalor and related conditions, two hypotheses were proposed. The rate of squalor was expected to be higher in areas where deprivation was more severe and in individuals with lower well-being.

Several significant differences, such as deprivation, well-being measures, household income and general health, were identified when the individuals who completed the physical survey were initially separated into those who were living in squalor and those who were not. However, due to the difference in the sample sizes of the two groups, it was not possible to confidently state which of the factors were responsible for these differences. The final squalor and non-squalor study groups were identified by PSM, allowing for the independent variables of deprivation and well-being to be investigated while controlling other factors.

Local deprivation had not previously been considered in the squalor literature. Therefore, this represented a new approach in the squalor evidence base. The mean data suggested that local deprivation was more severe in squalor households than in non-squalor households, though effect size was small. However, the regression analysis also supported deprivation as a key variable, showing it to be a predictor of whether an individual lived in squalor. These findings support the first hypothesis and suggest that local deprivation is a significant factor in the household conditions being of significantly high risk. Although no deprivation measure has previously been taken in the squalor literature, other studies have looked at the relationship between deprivation and a number of other mental health conditions [28, 31, 32, 60, 61]. This evidence base suggests that there is a higher risk of mental health problems in areas that are more deprived, and this appears to have been supported by the present study, which found an increased risk of squalor in more deprived areas. Although the finding of a significant impact of deprivation is consistent with previous research, it also builds on these findings. It demonstrates that deprivation is a key factor in the risks associated with the domestic hygiene, pests and refuse in a household. This is similar to the findings of the studies on self-neglect [31, 32]. However, self-neglect is not limited solely to individuals living in squalor conditions. Furthermore, Day et al. [31] and Lauder and Roxburgh [32] only considered individuals reported to be self-neglecting, whereas this study investigated squalor in the general population. Local deprivation appears to be related to both squalor and SN. However, whether there is a relationship between deprivation and Hoarding Disorder (HD) is less clear. HD has been linked to early-life material deprivation [62], but no research has yet investigated HD and local deprivation. Nonetheless, research does suggest that hoarding behaviours are more prevalent where income is lower [63], suggesting that a relationship between squalor, SN, HD and local deprivation could be present and may warrant further investigation.

It is likely that there is a bi-directional causal effect in the relationship between squalor and deprivation, in that living in squalor appears to be a product of local deprivation and that local deprivation will be added to by those houses that are squalid [64]. Furthermore, individuals living in squalor, or with related conditions, may be more likely to move into areas of high deprivation as their mental health conditions may increase the risk of legal issues and evictions [65–67], making it more difficult for them to find accommodation and therefore, more likely to live in deprived areas. Nonetheless, the suggestion of a bi-directional effect is speculative and includes the strong caveat that the cross-sectional nature of the data reported here limits interpretation of causality.

Mental health has been shown to be a significant concern for individuals who live in squalid conditions. Most notably, conditions including dementia, psychosis and addiction [7, 11, 24]. Measures of mental well-being have been shown to be associated with each of these conditions [36, 39, 68] and also to predict the risk of common mental disorders and to be strongly associated with self-reported mental health [69–72]. In the current study, poorer well-being was found in those living in squalor; however, once variables were controlled using PSM, there was no significant difference between the well-being of those living in squalor and those not living in squalor. Therefore, the second hypothesis, that individuals with lower well-being are more likely to be living in squalor, was not supported. Although this goes against much of the literature on squalor, it may be that well-being was measured using a relatively simple four-question measure that has not received significant validation. A more extensive and reliable measure that has been designed to produce a total score may have been more sensitive to differences in well-being. Alternatively, individuals living in squalor often have a poor awareness of their surroundings, therefore seeing little to be concerned about and this mitigates against feeling upset by their context [8, 19, 41]. If this was the case in the present sample, then the well-being of the participants was unlikely to be affected by their living environment and would not be represented in the self-report scale used in the EHS.

### Future research

Research needs to identify whether the relationship between deprivation and squalor is a causal link, or driven by a third, unknown variable, or whether there is bi-directional causality. Furthermore, as deprivation refers to the local neighbourhood, this suggests that community-level indicators, such as social cohesion and ethnic fractionalisation, may also be worth investigating. Although not the focus of this research, the preliminary analysis suggested that factors such as household income, whether the home was rented or owned, and who was living in the property, all differed significantly between those living in squalor and those who were not. Additional investigations would be necessary to identify whether these were still significant when other factors are controlled but could potentially suggest further household factors that could help to identify those at risk of squalor. Future research should also investigate the mental health of people in squalor using reliable methods. Use of diagnostic interviewing would add much to the literature on squalor. Finally, research could record the degree to which the resident themself rates the condition of the home and then compare this to objective ratings. Measures of dissociation with people living in squalor would be useful.

### Limitations

Due to the nature of squalor, which occurs in less than 1% of the population, a large sample is required to identify a suitably large group of individuals living in squalor. This is made more difficult as many individuals living in squalor will not consent to having a physical survey completed on their property, reducing the number of participants further. Therefore, any survey of the general population will show a significant difference between the squalor and non-squalor groups. In the present study, PSM was used to create a matched control group to compare with the squalor individuals. However, this did mean the loss of significant amount of data. Although this approach allowed for a more valid comparison of squalor and non-squalor individuals, it also meant that a large proportion of the overall sample was discarded.

In this study, squalor was ascertained from a domestic hygiene rating taken as part of a physical survey. Therefore, the dependent variable was not based on a validated measure of squalor, but instead a surveyor’s judgement of the risk associated with domestic hygiene, pests and refuse. Consequently, it is not possible to identify how the rating of squalor in this study compares to that used in other research, including those that have used squalor measures such as the Living Conditions Rating Scale (LCRS) or Environmental Cleanliness and Clutter Scale (ECCS) to provide an indication of the level of squalor present in a dwelling [7, 41, 73]. Furthermore, unlike these scales, the measure used in the EHS had only 4 levels and most individuals (over 99%) were rated as ‘average’. This provided limited detail as to the conditions in most households and only allowed for a basic identification of squalor or no squalor. There were also limitations surrounding the use of well-being as a variable and the use of the ONS-4 as a measure. A more accurate understanding of the mental health of the individuals living in squalor could have been achieved had the survey used a more extensive and validated measure, such as the Kessler K10 [74]. Finally, well-being measures were only assessed with the individual who completed the survey. Other adults in the household who were not present when the survey was completed would be missing data in this area.

## Conclusions

This study represents a unique approach to squalor research. It is the first study to consider squalor in a sample from the general population using rigorous methods. The use of this population has also enabled squalor to be investigated across the adult age range, as it was not limited to those who had become known to services, or those only in older adult age range. It is also the largest sample of squalor individuals investigated in any published study and the first to create a matched control group using PSM to identify individuals with other similar characteristics. Where squalor cases are carefully matched to non-squalor cases then key differences emerge in terms of the role of local deprivation, and this being more important than psychological aspects. This is important information of the potential to include squalor in terms of a neighbourhood effect [75] and this clearly requires more research using the best and most reliable methods.

## Data Availability

The datasets supporting the conclusions of this article are available in the UK Data Service repository, ukdataservice.ac.uk.The following datasets were analysed in this research:• 8387– English Housing Survey 2016: Housing Stock Data: Special Licence Access• 8851– English Housing Survey 2018: Housing Stock Data: Special Licence AccessRestrictions apply to the availability of these datasets, which were used under license for the current study, and so are not publicly available. Completion of an application was required to gain access.

## Abbreviations

BAME: Black, Asian, and Minority Ethnic
DS: Diogenes Syndrome
ECCS: Environmental Cleanliness and Clutter Scale
EHS: English Housing Survey
IMD: Index of Multiple Deprivation
LCRS: Living Conditions Rating Scale
ONS-4: Office of National Statistics Subjective Well-Being Questions
PSM: Propensity Score Matching
SDS: Severe Domestic Squalor
SMD: Standard Mean Difference
SN: Self-neglect
VR: Variance Ratio
